# Bushen Huoxue prescription inhibits ferroptosis of HUVEC cells by regulating FABP1-mediated lipid metabolism

**DOI:** 10.3389/fphar.2025.1636507

**Published:** 2025-09-26

**Authors:** Jingyi Chen, Xiaoying Song, Jiajie Xie, Danfei Lou

**Affiliations:** Emergency Department, Shanghai Municipal Hospital of Traditional Chinese Medicine, Shanghai University of Traditional Chinese Medicine, Shanghai, China

**Keywords:** Bushen Huoxue prescription, HUVEC cells, ferroptosis, FABP1, lipid metabolism

## Abstract

**Introduction:**

Atherosclerosis (AS) is a chronic inflammatory disease with vascular homeostasis imbalance, whose main characteristics are plaque formation and lipid accumulation. In traditional Chinese medicine, AS is associated with kidney deficiency and blood stasis. Bushen Huoxue prescription (BSHXP), based on the principle of tonifying the kidney and promoting blood circulation, has been widely used in clinical practice. However, its effects on AS are still indeterminate. This study aims to explore the effects and related mechanisms of BSHXP on oxidized low-density lipoprotein (ox-LDL)-induced HUVEC cells.

**Methods:**

Ox-LDL-induced HUVEC injury models were established by the ox-LDL incubation for 24 h, followed by BSHXP (12.5, 25, and 50 μg/mL) treatment. Cell viability was measured by MTT assay. Lipid metabolism was assessed by the Nile Red and Oil Red O staining. Inflammatory cytokines and ferroptosis markers were determined. Apoptosis was detected by Annexin V-FITC/PI staining.

**Results:**

UHPLC-Q/Exactive identified 8 main metabolites. Network pharmacology predicted 12 core metabolites, 10 hub targets, and key pathways related to lipid metabolism and ferroptosis. BSHXP regulated lipid metabolism by reducing FABP1 and SREBP2 expression and decreasing lipid droplet accumulation (*P* < 0.05). BSHXP inhibited ferroptosis by lowering Fe^2+^, ROS, ACSL4, and 4-HNE levels while increasing GSH, GPX4, and SLC7A11 (*P* < 0.05). FABP1 knockdown had similar effects, while FABP1 overexpression and ferroptosis inducer Erastin reversed the effects of BSHXP (*P* < 0.05). BSHXP also reduced IL-1β, IL-6, MCP-1, VCAM-1, and apoptosis (*P* < 0.05).

**Conclusion:**

BSHXP alleviates ox-LDL-induced HUVEC injury by inhibiting ferroptosis through FABP1-mediated lipid metabolism regulation.

## Graphical Abstract

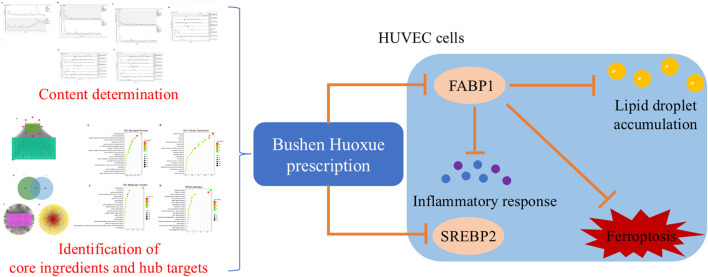

## 1 Introduction

According to statistics, cardiovascular disease is currently the leading cause of death in the world, including ischemic cardiomyopathy, acute myocardial infarction, and atherosclerosis (AS) (Crea, 2022). AS is a chronic disease involving endothelial cells, smooth muscle cells, and macrophages, whose primary inducements include endothelial injury, inflammatory response, and lipid deposition (Weber and Noels, 2011). The increase of inflammatory factors in blood vessels and the lipid accumulation in the vascular lumen lead to the formation of smooth muscle foam cells and macrophage foam cells, resulting in the vascular endothelial cell dysfunction and inflammatory reaction to form atherosclerotic plaques in the arterial wall (Getz and Reardon, 2012; Moore and Tabas, 2011). Vascular endothelial injury is the initial link to induce atherosclerosis. In recent years, increasing evidence has suggested that metabolic disorders in vascular endothelium, such as glycolysis, fatty acid oxidation, and abnormal glutamine and lipid metabolisms, are important triggers for endothelial cell dysfunction (Rohlenova et al., 2018). In addition, it was found that iron accumulation caused endothelial cell oxidation and an inflammatory reaction to produce intracellular ROS and facilitate lipid peroxidation, thereby further increasing the instability of atherosclerotic plaque (Xu, 2019). Although previous studies have affirmed that abnormal lipid metabolism and ferroptosis were related to the pathological process of AS, the specific related mechanisms are still unclear (Zhang et al., 2024a; Gkretsi et al., 2011). Therefore, this study investigated the pathological mechanisms of AS to facilitate its clinical treatment.

The main mechanism of ferroptosis is the loss of dynamic balance between the oxidative and antioxidant systems, whose biological characteristics are the increase in ROS and membrane lipid peroxidation because of the accumulation of iron ions and antioxidant dysfunction (Li et al., 2020). Fatty acid binding protein 1 (FABP1) is one of the fatty acid binding proteins, whose dominant biological function is to participate in fatty acid uptake, transport, fatty acid esterification, and the synthesis of phospholipids (Gaffar and Aathirah, 2023). More than that, FABP1 is also involved in regulating oxidative stress, which is caused by its methionine and cysteine residues (Le et al., 2018). It was reported that silencing FABP1 expression mitigated oxidative stress in mice with nonalcoholic fatty liver disease (Mukai et al., 2017). In addition, previous studies suggested that FABP5 was a functional biomarker and indicator of ferroptosis (Sun et al., 2024; Peng et al., 2024). However, the regulatory effects of FABP1 on ferroptosis are still unclear. Therefore, this study investigated whether regulating FABP1-mediated lipid metabolism alleviated ferroptosis in AS.

The role of traditional Chinese medicine (TCM) in treating AS with characteristics of *Mai Bi* (vascular impediment), dizziness, *Xiong Bi* (chest obstruction with pain), *Zhong Feng* (stroke), and headache has been gradually recognized (Liu et al., 2022a). According to TCM theory, the fundamental pathogenesis of AS involves kidney essence deficiency (*Shen Jing Xu Shuai*) as the inherent characteristic, and phlegm and blood stasis (*Tan Yu Nei Zu*) as the manifestation. The therapeutic principle of tonifying the kidney and promoting blood circulation (*Bu Shen Huo Xue*) has long been adopted by TCM practitioners as an effective strategy in the clinical management of AS. The botanical drug formula Bushen Huoxue prescription (BSHXP), based on this theory, is used for the treatment of AS-related disorders twice a day for 12 weeks with positive efficacy in our previous study (Xinyue et al., 2025). BSHXP consists of eight botanical drugs, including Radix rehmanniae praeparata [Scrophulaceae; Rehmannia glutinosa Libosch root], *Polygonatum sibiricum* F. Delaroche [Asparagaceae; Polygonatum sibiricum radix et rhizoma], *Lycium chinense* Miller [Solanaceae; Lycium chinense fruit], *Panax notoginseng* (Burkill) F. H. Chen ex C. H. Chow [Araliaceae; Panax notoginseng radix et rhizoma], *Ligusticum chuanxiong* Hort. [Umbelliferae; Ligusticum sinense rhizoma], *Salvia miltiorrhiza* Bunge. [Lamiaceae; Salviae miltiorrhizae radix et rhizoma], *Trichosanthes kirilowii* Maxim. [Cucurbitaceae Juss.; Trichosanthis pericarpium], and *Crataegus pinnatifida* Bge. [Rosaceae; Crataegus pinnatifida fruit]. Among them, *P. notoginseng* (Burkill) F. H. Chen ex C. H. Chow, *L. chuanxiong* Hort., and *S. miltiorrhiza* Bunge., which promote blood circulation, remove blood stasis, and have anti-inflammatory effects (Yang et al., 2022; Qi et al., 2024; Chen and Chen, 2017). The previous studies affirmed that the extracts of Radix rehmanniae praeparata and *P. sibiricum* F. Delaroche, as well as *L. chinense* Miller polysaccharide, all could regulate lipid metabolism (Luo et al., 2024; Wang et al., 2022a; Wu et al., 2024a). Moreover, Pericarpium trichosanthis polysaccharide and *C. pinnatifida* Bge. also effectively regulated cholesterol, glucose, and lipid metabolism (Jing et al., 2024; Wang et al., 2011). In addition, it was reported that Radix rehmanniae praeparata could regulate intracellular iron homeostasis, and *L. chinense* Miller polysaccharide and *S. miltiorrhiza* Bunge. effectively alleviated ferroptosis (Zhang et al., 2024b; Yang et al., 2024; Wu et al., 2024b). However, the effects of BSHXP in alleviating AS by regulating lipid metabolism to inhibit ferroptosis are still unclear.

Against the above backgrounds, this study aimed to investigate whether BSHXP regulated FABP1-mediated lipid metabolism to alleviate ferroptosis, ultimately mitigating oxidized low-density lipoprotein (ox-LDL)-induced human umbilical vein endothelial cells (HUVECs) injuries, providing a theoretical basis for AS clinical treatment using BSHXP.

## 2 Materials and methods

### 2.1 Preparation of BSHXP

After all crude botanical drugs, including Radix rehmanniae praeparata (30 g), *P. sibiricum* F. Delaroche (30 g), *L. chinense* Miller (30 g), *P. notoginseng* (Burkill) F. H. Chen ex C. H. Chow (10 g), *L. chuanxiong* Hort. (20 g), *S. miltiorrhiza* Bunge. (30 g), *T. kirilowii* Maxim. (60 g), and *C. pinnatifida* Bge. (30 g), were identified by the director Xu Yanfeng of the pharmacy department of our hospital, they were soaked in 2000 mL of water for 2 h. After that, they were boiled with water and simmered at low heat for 30 min. Then, they were filtered to collect the decoction. Meanwhile, after the raw residue was boiled with 2000 mL of water again, the second decoction was collected. After mixing the two decoctions (approximately 2,500 mL) and concentration, they were freeze-dried using a lyophilizer (LAB1-50, Biocool, Beijing, China). In short, the decoction was pre-frozen at −50 °C for 5 h before the vacuum drying. Then, the dryer was heated and finally fixed at −40 °C for 72 h to prepare lyophilized powder. Finally, a total of approximately 560 mg lyophilized powder of BSHXP was obtained and used for subsequent studies. All voucher specimens were deposited in our department.

### 2.2 Preparation of standard stock solution mixtures

The standards of Tanshinone IIA (purity: 99.78%, Lot., HY-N0135), salvianolic acid B (purity: 99.92%, Lot., HY-N1362), ferulic acid (purity: 99.97%, Lot., HY-N0060), Ginsenoside Rg1 (purity: 99.91%, Lot., HY-N0045), Ginsenoside Rb1 (purity: 99.28%, Lot., HY-N0039), Rehmannia glutinosa D (purity: 99.56%, Lot., HY-N0025), betaine (purity: 99.84%, Lot., HY-N0710), and chlorgenic acid (purity: 99.53%, Lot., HY-N0055) were weighed each 1-2 mg, and dissolved in methanol (HPLC-grade, Lot., A456-4, Fisher Scientific, Pennsylvania, USA) to obtain the stock solutions of each reference substance. All standards were bought from MedChemExpress (New Jersey, USA). Take each reference substance stock solution and configure it into a mixed reference substance solution with a concentration of 1 μg/mL. All samples were stored at 4 °C until analysis.

### 2.3 Preparation of samples

After accurately weighing 100 mg lyophilized powder of BSHXP, they were dissolved in accurately measured 1 mL ultrapure water, and centrifuged at 12,000 rpm at 4 °C for 15 min. After accurately absorbing 10 μL supernatant, the ultrapure water was added to dilute to 100 times. Then, the 80 μL of diluent was accurately absorbed and added to the liquid phase vial for sample loading analysis.

### 2.4 UHPLC-Q/exactive analytical method

The chromatographic separation was achieved on an ACQUITY UPLC® HSS T3 column (2.1 mm × 100 mm, 1.8 μm, Waters Corporation, Milford, MA, United States) tandem with a guard column using a Thermo Scientific™ Q Exactive™ Quadrupole-Orbitrap Mass Spectrometer system (Thermo Fisher Scientific Inc., Grand Island, NY, United States). The UPLC system consisted of a Thermo Scientific Dionex Ultimate 3000 Series RS pump coupled with a Thermo Scientific Dionex Ultimate 3000 Series TCC-3000 RS column compartments and a Thermo Fisher Scientific Ultimate 3000 Series WPS-3000 autosampler controlled by Chromeleon 7.2 Software. The cooling autosampler was set at 10 °C and protected from light, and the column heater was set at 45 °C. A gradient elution programmer was used for chromatographic separation with mobile phase C (0.1% formic acid (HPLC-grade, purity >99%, CAEQ-4-000,313-0050, CNW Technologies, Bayern, Germany) in water) and mobile phase D [acetonitrile (HPLC-grade, Lot., A996-4, Fisher Scientific, Pennsylvania, United States)] mixed as follows: 0–12.0 min, 5% D - 95% D; 12.01–14.0 min, 95% D; 14.01–16 min, 5% D. The flow rate of the mobile phase was set at 0.3 mL/min, and the injection volume was 3 μL.

A Thermo Scientific™ Q-Exactive™ Quadrupole-Orbitrap Mass Spectrometer system (Thermo Fisher Scientific Inc., Grand Island, USA) connected to the UPLC system via a heat electrospray ionization interface and controlled by Xcalibur 4.1 software was used for data capture and analysis. MS/MS was operated in positive and negative mode using the following operating parameters: capillary temperature, 320 °C; Spray voltage, +3.5 KV/-2.8 KV; aux gas volume flow rate, 13 L/min; sheath gas flow rate, 80 L/min; aux gas heating temperature, 350 °C; resolution, 70,000; scan range, 80∼1,200 m/z.

### 2.5 Identifying active metabolites and targets of BSHXP

Based on the limiting conditions of OB ≥ 30% and DL ≥ 0.18, the Traditional Chinese Medicine Systems Pharmacology and Analysis Platform (TCMSP) database (http://tcmspw.com/tcmsp.php) was applied to screen the active metabolites of various botanical drugs in BSHXP. After retrieving the SMILES formula of active metabolites in the PubChem database (https://pubchem.ncbi.nlm.nih.gov/), the potential targets of active metabolites were predicted by the Swiss Target Prediction (http://www.swisstargetprediction.ch). Finally, after transforming target protein names into corresponding gene names using the Uniprot database (https://www.uniprot.org/), an active ingredients-targets network was established using the Cytoscape 3.8.0 software.

### 2.6 Prediction of AS targets and identification of core metabolites for BSHXP

After inputting the keyword “Atherosclerosis,” the disease targets of AS were screened through the GeneCards database (https://www.genecards.org/). After removing duplicate targets, the intersection of drug and disease targets was visualized in a Venn diagram. An active ingredients-targets network was established again to select the core metabolites with the top 12 Degree value.

### 2.7 Construction of protein-protein interaction (PPI) networks and identification of hub targets

Based on the STRING database (https://string-db.org/), the PPI networks were established by selecting the “Multiple Proteins” tool, defining the Organism as “*Homo Sapiens*,” setting high confidence (0.900), and hiding the free nodes. By using the Cytoscape 3.8.0 software, the PPI networks were adopted for further topological analysis to select hub targets with the top 10 Degree values.

### 2.8 Gene ontology (GO) and kyoto encyclopedia of genes and genomes (KEGG) analysis

The GO and KEGG enrichment analysis were accomplished using the DAVID database (https://david.ncifcrf.gov/). After setting the threshold to *P* < 0.05 and sorting by the enrichment count, the enrichment results were visualized in a bubble chart.

### 2.9 Cell culture and cell modeling

The HUVEC cell line was bought from Immocell (Xiamen, China). The HUVEC cells were cultured in the DMEM culture medium (Lot. 11965118, Gibco, Grand Island, United States) supplemented with 10% fetal bovine serum (Lot. FSD500, Excell Bio, Shanghai, China), and 1% penicillin-streptomycin (Lot. C0222, Beyotime, Shanghai, China) with surroundings of 37 °C, 5% CO_2_, and 95% humidity environment. Based on the previous study, this study used ox-LDL (100 μg/mL, Lot. 20605ES05, Yesen, Shanghai, China) to irritate HUVEC cells for 24 h to imitate AS *in vitro* (Men et al., 2024).

### 2.10 Cell experiment protocol and cell transfection

To determine the effects of BSHXP on lipid metabolism, HUVEC cells were stochastically separated into five groups, namely, the control, ox-LDL, BSHXP-L, BSHXP-M, and BSHXP-H groups. Apart from the control group, HUVEC cells in other groups were treated with ox-LDL (100 μg/mL) for 24 h (Gong et al., 2019). In addition, HUVEC cells in the last three groups were treated with 12.5, 25, and 50 μg/mL BSHXP lyophilized powder dissolved in DMEM culture medium for 24 h, respectively. To determine the effects of regulating FABP1 on the ferroptosis of HUVEC cells, they were stochastically divided into four groups, namely, the control, ox-LDL, sh-NC, and sh-FABP1 groups. HUVEC cells were normally cultured in the control group and were treated with ox-LDL in the other groups. In addition, HUVEC cells in the last two groups were respectively transfected with sh-NC (5′-CAC​CGT​TCT​CCG​AAC​GTG​TCA​CGT​TTC​AAG​AGA​ACG​TGA​CAC​GTT​CGG​AGA​ATT TTT​TG-3′) and sh-FABP1 (5′-AGC​AAA​ACC​TAG​TGA​AAC​CTG​TTC​AAG​AGA​CAG​GTT​TCA​CTA​GGT​TTT​GCT-3′) for 72 h using Lipofectamine 3000 (Lot. L3000015, Thermo Fisher Scientific, Waltham, USA). To investigate whether BSHXP alleviated HUVEC cell injuries by regulating FABP1, they were stochastically divided into five groups, namely, the control, ox-LDL, BSHXP, BSHXP + oe-NC, and BSHXP + oe-FABP1 groups. HUVEC cells were normally cultured in the control group and were treated with ox-LDL in the other groups. Before being treated with 50 μg/mL BSHXP, HUVEC cells in the last two groups were respectively transfected with oe-NC (5′-GAT​CCA​AAA​AAT​TCT​CCG​AAC​GTG​TCA​CGT​TCT​CTT​GAA​ACG​TGA​CAC​GTT​CGG AGA​AC-3′) and oe-FABP1 (5′-AAG​CAA​AAC​CTA​GTG​AAA​CCT​TTC​AAG​AGA​AGG​TTT​CAC​TAG​GTT​TTG​CTT-3′) for 72 h. To investigate whether BSHXP inhibited ferroptosis by regulating FABP1, HUVEC cells were stochastically divided into five groups, namely, the control, ox-LDL, BSHXP, BSHXP + oe-FABP1, and BSHXP + erastin groups. The treatments of HUVEC cells in the first four groups were the same as mentioned above. Apart from being treated with ox-LDL and BSHXP for 24 h, HUVEC cells in the last group were also treated with erastin (10 μM, ferroptosis inducer, Lot. HY-15763, MedChemExpress, Princeton, USA) for 24 h (Lv et al., 2023). All transfectants were synthesized by Sangon (Shanghai, China).

### 2.11 MTT assay

The MTT assay was adopted in this study to determine the safe concentrations of BSHXP on HUVEC cells. In short, after HUVEC cells were respectively treated with BSHXP lyophilized powder dissolved in DMEM culture medium (0, 6.25, 12.5, 25, 50, and 100 μg/mL) for 24 h, 10 μL MTT agent (0.5 mg/mL, Lot. C0009S, Beyotime, Shanghai, China) was supplemented and incubated for 4 h. Then, after supplementing with 100 μL dimethyl sulfoxide, the optical density (OD) values of HUVEC cells at 570 nm were detected by a Multiskan FC microplate reader 357-714018 (Thermo Fisher Scientific, Waltham, USA). The cell viability of HUVEC cells was calculated based on their OD values.

### 2.12 Nile red staining

After finishing corresponding treatments, the HUVEC cells were fixed with 4% paraformaldehyde (Lot. P0099, Beyotime, Shanghai, China). Then, the HUVEC cells were successively stained with 1 mL staining solution composed of Nile red reagent (Lot. C2051S, Beyotime, Shanghai, China) and the Hoechst 33,342 solution (Lot. C1029, Beyotime, Shanghai, China) for 20 min in the dark. The stained results were observed using a BZ-H4XD fluorescence microscope (Keyence, Osaka, Japan), among which lipid droplets exhibited red fluorescence and cell nuclei displayed blue fluorescence. The Image-Pro Plus 6.0 software (Media Cybernetics Inc., Rockville, USA) was employed to quantify the fluorescence intensity to evaluate the lipid droplet content of HUVEC cells.

### 2.13 Oil red O staining

After finishing corresponding treatments, the HUVEC cells were fixed with oil red O fixed liquid. Then, they were washed with 60% isopropanol for 30 s. Next, after HUVEC cells were stained by the oil red O dye solution (Lot. C0157S, Beyotime, Shanghai, China) for 30 min, they were counterstained by Mayer’s hematoxylin solution (Lot. PR30004, Proteintech, Wuhan, China) for 15 min. Finally, the stained results were recorded by an Axio Imager M1 optical microscope (Zeiss, Oberkochen, Germany).

### 2.14 Intracellular Fe^2+^ and glutathione levels detection

The colorimetric method was applied to detect the intracellular Fe^2+^ level. In short, after finishing the corresponding treatments, the HUVEC cells were collected, resuspended in 250 μL lysis solution, and cracked at 4 °C for 30 min. According to the instructions of the cell ferrous iron colorimetric assay kit (Lot. E-BC-K881-M, Elabscience, Wuhan, China), the solutions in standard wells, measuring wells, and control wells were separately prepared. The intracellular Fe^2+^ level was determined based on the OD values at 593 nm detected by the microplate reader. The operation processes for detecting intracellular glutathione (GSH) level were similar to those for intracellular Fe^2+^ level. The differences were that the reduced GSH detection kit (Lot. ZC-S0329, Zcibio, Nanjing, China) was employed, the intracellular GSH level was determined based on the calibration curve method, and the OD values were detected at 412 nm.

### 2.15 ELISA

After finishing the corresponding treatments, the HUVEC cell supernatant was gathered. The interleukin-1β (IL-1β), interleukin-6 (IL-6), monocyte chemoattractant protein-1 (MCP-1), and vascular cell adhesion molecule-1 (VCAM-1) levels in the supernatant were respectively determined using corresponding human ELISA kits based on the producer’s instructions, among which the human IL-1β ELISA kit (Lot. PI305), human IL-6 ELISA kit (Lot. PI330), human CCL2/MCP-1 ELISA kit (Lot. PC130), and human VCAM-1/CD106 ELISA kit (Lot. PV955) were all bought from Beyotime (Shanghai, China).

### 2.16 Flow cytometry

To determine the apoptosis rate of HUVEC cells, after collecting HUVEC cells with corresponding treatments, they were successively stained by 5 μL Annexin V-FITC and PI (Lot. C1062L, Beyotime, Shanghai, China) in the dark for 20 min. To determine the ROS level of HUVEC cells, after finishing corresponding treatments, HUVEC cells were immersed in 1 mL DCFH-DA (10 µM) diluted by serum-free DMEM for 30 min in the dark. After finishing the above staining, the HUVEC cells were collected to determine the apoptosis rate and ROS level of HUVEC cells using an Attune NxT flow cytometer (Invitrogen, Carlsbad, United States).

### 2.17 QRT-PCR

After extracting total RNAs in HUVEC cells using the MiPure Cell/Tissue miRNA Kit (Lot. RC201, Vazyme, Nanjing, China), the total RNAs were reversely transcribed into cDNA and cDNA was amplified with the help of the miRNA 1st Strand cDNA Synthesis Kit (Lot. MR101, Vazyme, Nanjing, China) and miRNA Universal SYBR qPCR Master Mix (Lot. MQ101, Vazyme, Nanjing, China), respectively. The reverse transcription and cDNA amplification were performed in a CFX96 Touch 1855195 qRT-PCR instrument (Bio-Rad, Hercules, United States). The 2^−ΔΔCT^ approach was employed for the quantification of FABP1 expression level. The primer sequences of FABP1 and GAPDH are listed in Table 1, in which GAPDH is the internal reference.

**TABLE 1:** The primer sequences for qRT-PCR.

Primers	Sequence (5’→3′)	
FABP1	Forward	GAG​GGA​GCT​CTA​TTG​CCA​CC
	Reverse	TGG​ATC​ACT​TTG​GAC​CCA​GC
GAPDH	Forward	AAT​GGG​CAG​CCG​TTA​GGA​AA
	Reverse	GCG​CCC​AAT​ACG​ACC​AAA​TC

### 2.18 Western blot

After the total proteins in HUVEC cells were extracted and denatured in boiling water, they were loaded on the SDS-PAGE gel and transferred to the PVDF membranes. Then, after being blocked by skim milk, the membranes were immersed in the primary antibodies at 4 °C for one night, including anti-FABP1 (1: 1,000, Lot. #13368), anti-sterol regulatory element-binding protein 2 (SREBP2, 1: 200, Lot. PA5-88943), anti-solute carrier family 7 member 11 (SLC711A, 1: 1,000, Lot. ab307601), anti-glutathione peroxidase 4 (GPX4, 1: 500, Lot. ab125066), anti-acyl-coA synthetase long chain family member 4 (ACSL4, 1: 2000, Lot. ab155282), anti-4-Hydroxy-2-nonenal (4-HNE, 1: 3500, Lot. ab46545), and anti-GAPDH (1: 2000, Lot. bsm-33033M). Apart from the anti-FABP1, anti-SREBP2, and anti-GAPDH were respectively bought from Cell Signaling Technology (Danvers, United States), Thermo Fisher Scientific (Waltham, USA), and Bioss (Beijing, China), other antibodies were purchased from Abcam (Cambridge, UK). Finally, after incubating with the goat anti-mouse IgG H&L/HRP (1: 20,000, Lot. bs-0296G-HRP, Bioss, Beijing, China) for 30 min and being stained by ECL chemiluminescent substrate, the grayscale value of blots was quantified using the Image-Pro Plus 6.0 software.

### 2.19 Statistical analysis

All data produced from independently triplicate experiments were exhibited as mean ± standard deviation. Statistical significance was determined using Student’s t-test or one-way ANOVA, followed by Tukey’s *post hoc* test, with the help of GraphPad Prism 8.0.2 (La Jolla, California, USA). A p-value of less than 0.05 was considered statistically significant.

## 3 Results

### 3.1 Content determination of the eight main metabolites in BSHXP

**FIGURE 1:**
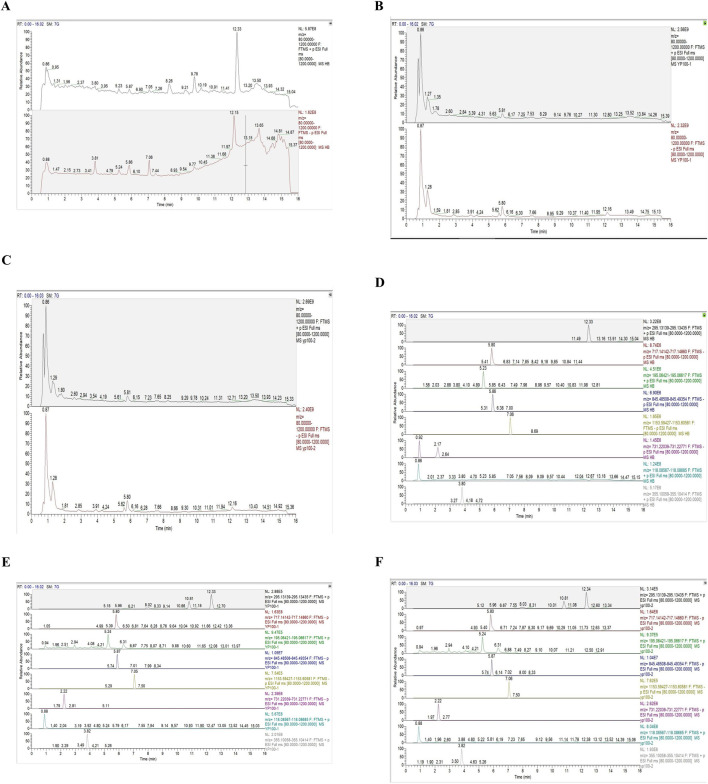
Representative SIM chromatograms and total ion flow chromatograms of a mixed standard solution and samples. The total ion flow chromatogram of a mixed standard solution **(A)**, sample 1 **(B)**, and sample 2 **(C)**. The SIM chromatograms of standards **(D)**, sample 1 **(E)**, and sample 2 **(F)** (Tanshinone IIA:12.33 min, salvianolic acid B:5.80 min, ferulic acid:5.23 min, Ginsenoside Rg1:5.86 min, Ginsenoside Rb1:7.06 min, Rehmannia glutinosa D:2.17 min, betaine:0.86 min, chlorgenic acid:3.80 min).

The main parameters for standards are shown in Table 2. Representative single ion monitoring (SIM) chromatograms and total ion flow chromatograms of a mixed standard solution and sample are shown in Figures 1A–F. The external standard one-point method was used to analyze BSHXP lyophilized powder samples. The analysis results of the BSHXP lyophilized powder are listed in Table 3. A total of 8 metabolites were detected in the BSHXP lyophilized powder (Tanshinone IIA, salvianolic acid B, ferulic acid, Ginsenoside Rg1, Ginsenoside Rb1, Rehmannia glutinosa D, betaine, and chlorgenic acid). Among them, the content of salvianolic acid B, Ginsenoside Rg1, Rehmannia glutinosa D, and betaine is higher.

**TABLE 2:** Main parameters for standards.

NO.	Name	Molecular formula	Theoretical molecular weight	[M+H]+	[M-H]-	[M+HCOO]-	Rt/min
1	Tanshinone IIA	C19H18O3	294.12505	295.13287	—	—	12.33
2	salvianolic acid B	C36H30O16	718.15284	—	717.14501	—	5.80
3	ferulic acid	C10H10O4	194.05736	195.06519	—	—	5.23
4	Ginsenoside Rg1	C42H72O14	800.49166	—	—	845.48931	5.86
5	Ginsenoside Rb1	C54H92O23	1,108.60239	—	—	1,153.60004	7.06
6	Rehmannia glutinosa D	C27H42O20	686.22639	—	—	731.22405	2.17
7	betaine	C5H11NO2	117.07843	118.08626	—	—	0.86
8	chlorgenic acid	C16H18O9	354.09453	355.10236	—	—	3.80

**TABLE 3:** The content of 8 metabolites in the BSHXP lyophilized powder (μg/mL).

NO.	Name	Sample 1	Sample 2	Average
1	Tanshinone IIA	0.28567	0.29159	0.28863
2	salvianolic acid B	1938.68389	1938.68389	1938.68389
3	ferulic acid	19.66075	19.66075	19.66075
4	Ginsenoside Rg1	140.76257	140.76257	140.76257
5	Ginsenoside Rb1	42.42683	42.42683	42.42683
6	Rehmannia glutinosa D	203.23581	203.23581	203.23581
7	betaine	464.24278	464.24278	464.24278
8	chlorgenic acid	26.99111	26.99111	26.99111

### 3.2 Identification of core metabolites and hub targets of BSHXP

**FIGURE 2:**
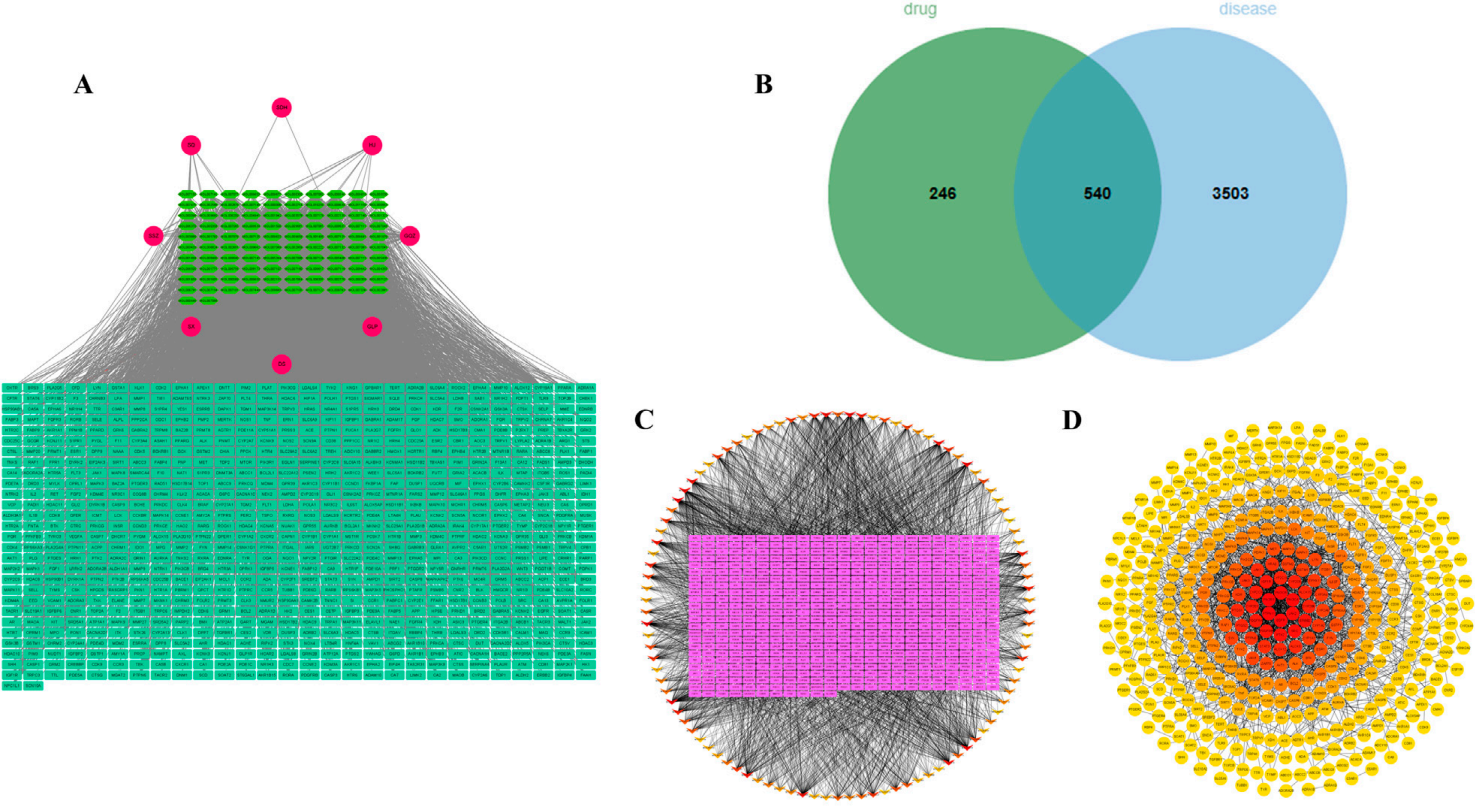
Identification of core metabolites and hub targets of BSHXP. **(A)** The network between 102 active metabolites and 786 targets of BSHXP. **(B)** The Venn diagram between 786 drug targets and 4043 disease targets. **(C)** The active ingredients-targets network was constructed based on 540 common targets. **(D)** The PPI network.

Based on the TCMSP and Swiss Target Prediction database, 102 active metabolites of BSHXP and 786 of their targets were predicted. The active ingredients-targets network was presented in Figure 2A. Based on 786 drug targets mentioned above and 4043 disease targets predicted by the GeneCards database, a Venn diagram was constructed and presented in Figure 2B, in which there were 540 common targets. According to the 540 targets mentioned above, the active ingredients-targets network was constructed again, among which the darker color represented the higher Degree value. As illustrated in Figure 2C, the core metabolites with top 12 Degree value were as follows, MOL006331 (4′,5-dihydroxyflavone), MOL002714 (baicalein), MOL001792 (liquiritigenin), MOL004941 ((2R)-7-hydroxy-2-(4-hydroxyphenyl) chroman-4-one), MOL000006 (luteolin), MOL000098 (quercetin), MOL007077 (sclareol), MOL007079 (tanshinaldehyde), MOL002135 (myricanone), MOL002151 (senkyunone), MOL001494 (mandenol), and MOL001495 (ethyl linolenate). After constructing the PPI network, hub targets with top 10 Degree values were screened and listed as follows: PIK3R1, PTPN11, EGFR, PIK3CA, PIK3CB, JAK2, SRC, PTK2, ERBB2, and PDGFRB (Figure 2D).

### 3.3 GO and KEGG enrichment analysis

**FIGURE 3:**
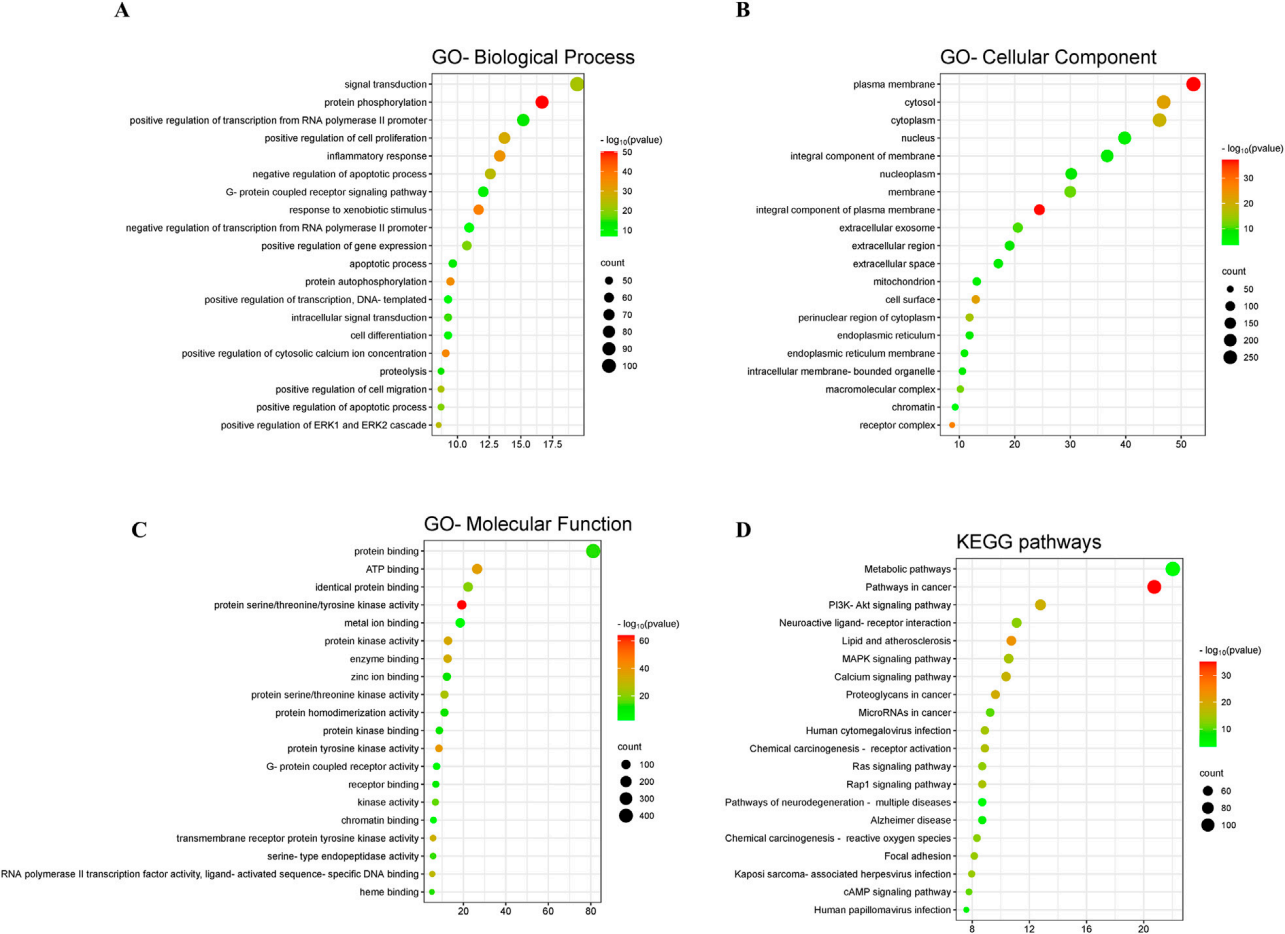
GO and KEGG enrichment analysis. The GO enrichment in the aspects of biological process **(A)**, cellular component **(B)**, and molecular function **(C)**. **(D)** The KEGG enrichment.

The GO enrichment results showed that, in the category of biological process, these targets were dominantly enriched in signal transduction, protein phosphorylation, positive regulation of transcription from RNA polymerase II promoter, positive regulation of cell proliferation, inflammatory response, negative regulation of apoptotic process, G-protein coupled receptor signaling pathway, response to xenobiotic stimulus, negative regulation of transcription from RNA polymerase II promoter, and positive regulation of gene expression (Figure 3A). In the category of cellular component, these targets were dominantly enriched in plasma membrane, cytosol, cytoplasm, nucleus, integral component of membrane, nucleoplasm, membrane, integral component of plasma membrane, extracellular exosome, and extracellular region (Figure 3B). In the category of molecular function, these targets were dominantly enriched in protein binding, ATP binding, identical protein binding, protein serine/threonine/tyrosine kinase activity, metal ion binding, protein kinase activity, enzyme binding, zinc ion binding, protein serine/threonine kinase activity, and protein homodimerization activity (Figure 3C). In addition, the KEGG enrichment results presented that these targets were primarily enriched in metabolic pathways, pathways in cancer, PI3K-Akt signaling pathway, neuroactive ligand-receptor interaction, lipid and atherosclerosis, MAPK signaling pathway, calcium signaling pathway, proteoglycans in cancer, microRNAs in cancer, and chemical carcinogenesis-receptor activation (Figure 3D).

### 3.4 BSHXP regulated lipid metabolism in HUVEC cells

**FIGURE 4:**
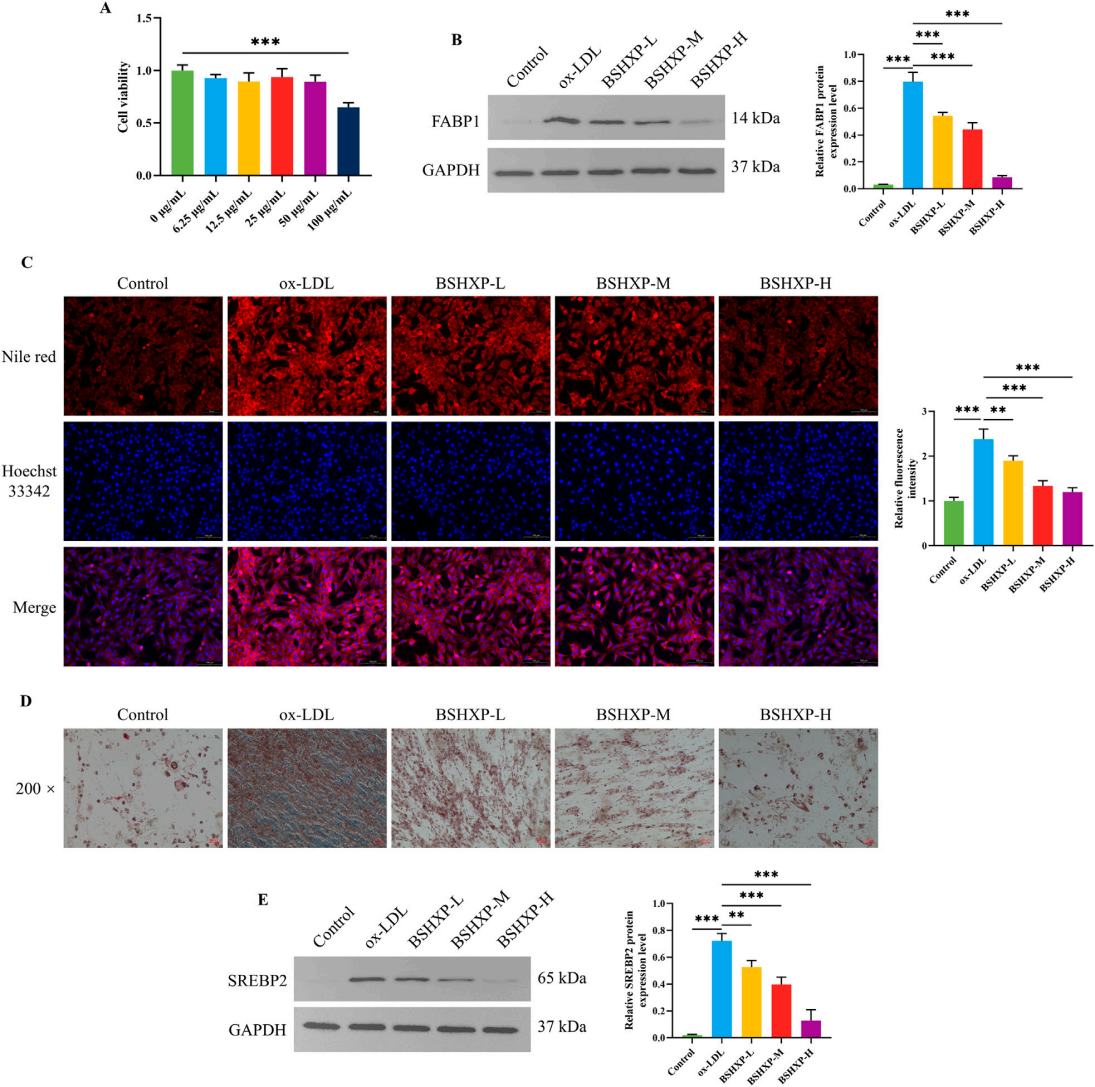
Effects of BSHXP on lipid metabolism of HUVEC cells. **(A)** The cell viability of HUVEC cells (n = 6). **(B)** The FABP1 protein expression level of HUVEC cells (n = 3). **(C)** The results of Nile red staining of HUVEC cells (n = 3). **(D)** The representative images of the oil red O staining of HUVEC cells with a magnification of ×200. **(E)** The SREBP2 protein expression level of HUVEC cells (n = 3). ^***/**^ *P* < 0.001/0.01.

First, we adopted the MTT approach to exclude the toxic dosage of BSHXP for HUVEC cells. The cell viability of HUVEC cells was prominently reduced when treated with 100 μg/mL BSHXP (Figure 4A). Therefore, the 100 μg/mL BSHXP was excluded, and 12.5, 25, and 50 μg/mL BSHXP were respectively considered the BSHXP-L, BSHXP-M, and BSHXP-H in subsequent studies. As the above results predicted that the PPAR signaling pathway was one enriched pathway of BSHXP, we subsequently investigated whether BSHXP regulated FABP1-mediated lipid metabolism (one regulatory molecule of the PPAR pathway) in HUVEC cells (Yan et al., 2023). As illustrated in Figure 4B, BSHXP-L, BSHXP-M, and BSHXP-H effectively reversed ox-LDL-induced elevation in the FABP1 protein expression level of HUVEC cells. In addition, BSHXP-L, BSHXP-M, and BSHXP-H prominently decreased the lipid droplet content (Figures 4C,D) and SREBP2 protein expression level (Figure 4E) of HUVEC cells in a dose-dependent way. The above results suggested that BSHXP regulated lipid metabolism in HUVEC cells, which might be related to mediating FABP1.

### 3.5 Knocking down FABP1 alleviated ferroptosis by regulating the lipid metabolism of HUVEC cells

**FIGURE 5:**
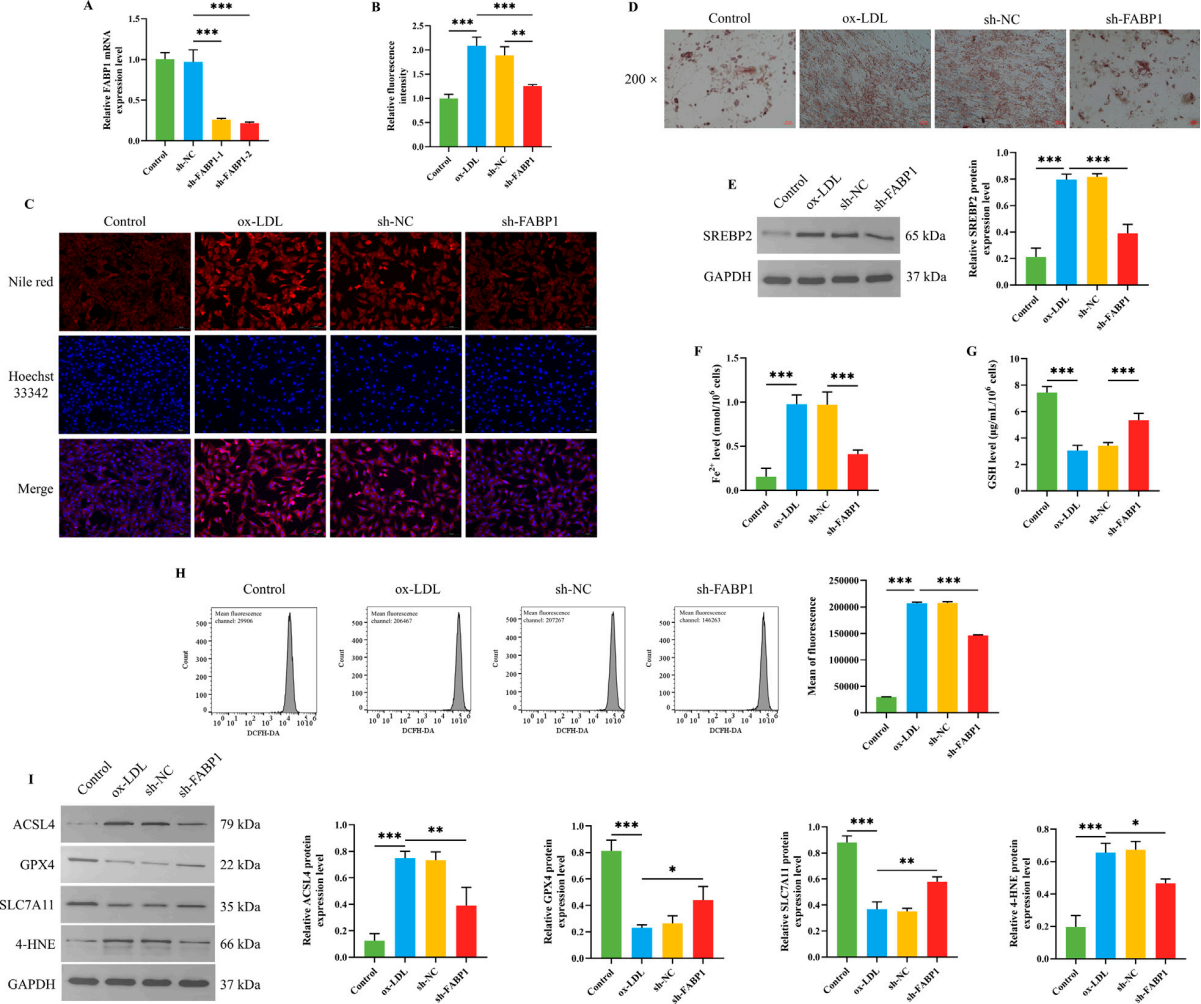
Effects of knocking down FABP1 on lipid metabolism and ferroptosis of HUVEC cells. **(A)** The FABP1 mRNA expression level of HUVEC cells (n = 3). **(B)** The statistical analysis for the fluorescence intensity of Nile red staining of HUVEC cells (n = 3). **(C)** The representative images for the Nile red staining of HUVEC cells. **(D)** The representative images of the oil red O staining of HUVEC cells with a magnification of ×200. **(E)** The SREBP2 protein expression level of HUVEC cells (n = 3). The Fe^2+^ **(F)** and GSH **(G)** levels in HUVEC cells (n = 6). **(H)** The ROS level of HUVEC cells was detected by a flow cytometer (n = 3). **(I)** The protein expression levels of ACSL4, GPX4, SLC711A, and 4-HNE in HUVEC cells (n = 3). ^***/**/*^ *P* < 0.001/0.01/0.05.

First, FABP1 mRNA expression level was determined to select the optimal transfectant. As presented in Figure 5A, the FABP1 mRNA expression level of HUVEC cells was dramatically downregulated after being transfected with sh-FABP1-1 and sh-FABP1-2, in which the FABP1 mRNA expression level was lower when being transfected with sh-FABP1-2. Therefore, the sh-FABP1-2 was used to knock down FABP1 expression in subsequent studies. As the above results indicated that FABP1 might be involved in the effects of BSHXP on lipid metabolism, we subsequently investigated the effects of knocking down FABP1 on the lipid metabolism of HUVEC cells. Knocking down FABP1 effectively reversed ox-LDL-induced elevation in the lipid droplet content (Figures 5B–D) and SREBP2 protein expression level (Figure 5E) of HUVEC cells. More interesting, as shown in Figures 5F–I, we discovered that knocking down FABP1 also substantially reversed ox-LDL-induced elevation in the Fe^2+^ and ROS levels as well as the ACSL4 and 4-HNE protein expression levels and reduction in the GSH level and GPX4 and SLC711A protein expression levels. The above results indicated that knocking down FABP1 mitigated ferroptosis by regulating the lipid metabolism of HUVEC cells.

### 3.6 BSHXP improved HUVEC cell injuries by inhibiting FABP1

**FIGURE 6:**
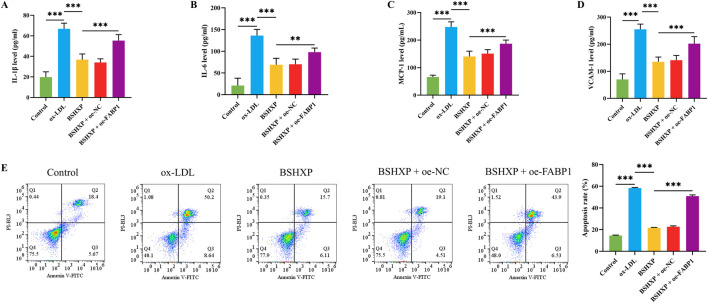
Effects of BSHXP on HUVEC cell injuries. The IL-1β **(A)**, IL-6 **(B)**, MCP-1 **(C)**, and VCAM-1 **(D)** levels of HUVEC cells (n = 6). **(E)** The apoptosis rate of HUVEC cells (n = 3). ^***/**^ *P* < 0.001/0.01.

As the above studies have confirmed that knocking down FABP1 reduced ferroptosis by regulating lipid metabolism, we next investigated whether BSHXP improved HUVEC cell injuries by regulating FABP1. Since the previous studies have demonstrated that AS belonged to the pathological process of chronic inflammation, the inflammation-related biomarkers were first detected (Wolf and Ley, 2019; Kong et al., 2022). BSHXP memorably downregulated IL-1β, IL-6, MCP-1, and VCAM-1 (Figures 6A–D) levels of HUVEC cells. Moreover, BSHXP also markedly reduced the apoptosis rate of HUVEC cells (Figure 6E) of HUVEC cells. In addition, overexpressing FABP1 effectively reversed the alterations of these indicators mentioned above. The above results revealed that BSHXP improved HUVEC cell injuries by suppressing FABP1.

### 3.7 BSHXP improved HUVEC cell injuries by inhibiting FABP1-mediated ferroptosis

**FIGURE 7:**
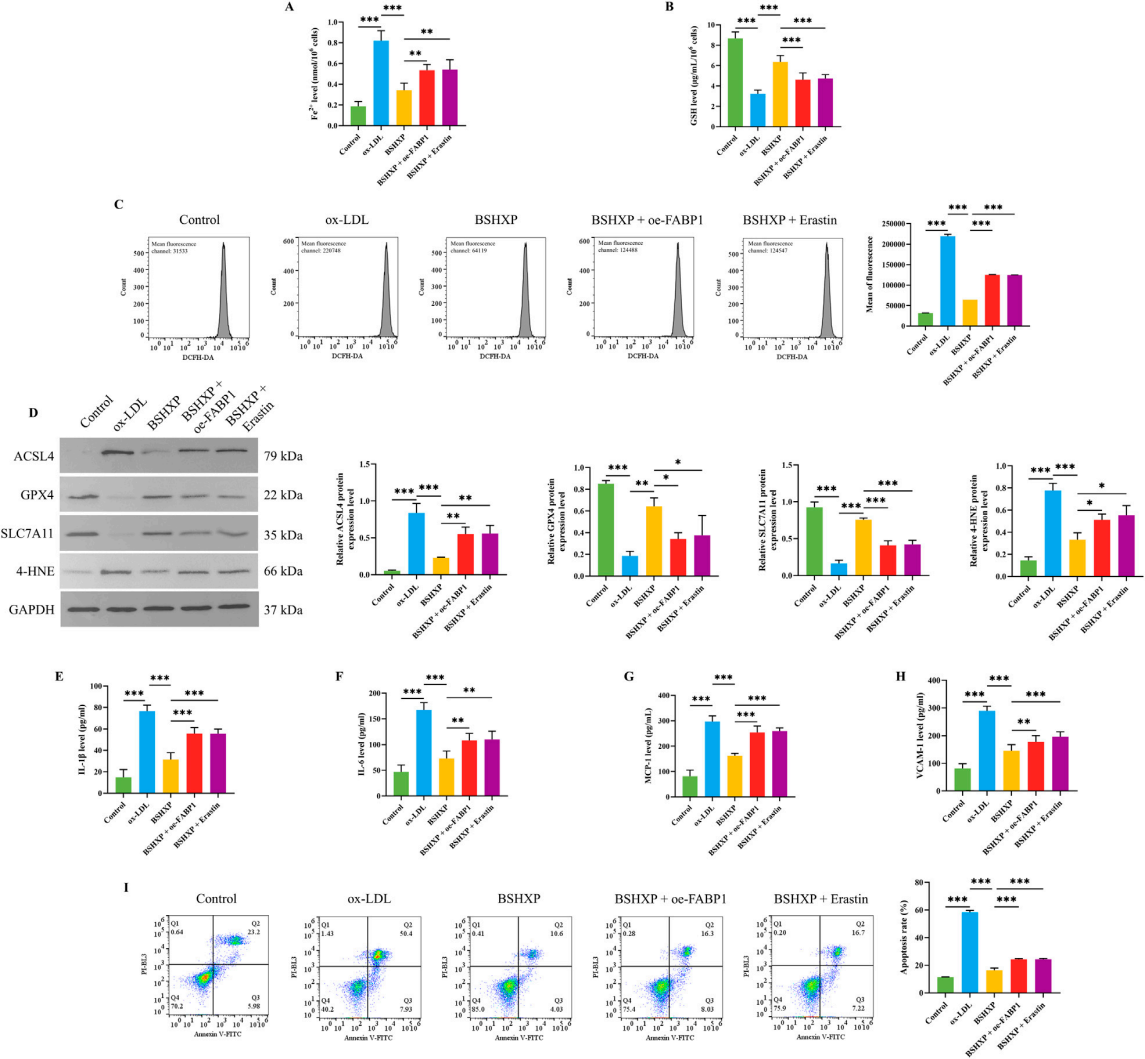
Effects of BSHXP on HUVEC cell injuries through regulating FABP1-mediated ferroptosis. The Fe^2+^ **(A)** and GSH **(B)** levels in HUVEC cells (n = 6). **(C)** The ROS level of HUVEC cells was detected by a flow cytometer (n = 3). **(D)** The protein expression levels of ACSL4, GPX4, SLC711A, and 4-HNE in HUVEC cells (n = 3). The IL-1β **(E)**, IL-6 **(F)**, MCP-1 **(G)**, and VCAM-1 **(H)** levels of HUVEC cells (n = 6). **(I)** The apoptosis rate of HUVEC cells (n = 3). ^***/**/*^ *P* < 0.001/0.01/0.05.

As the above studies have affirmed that BSHXP improved HUVEC cell injuries by inhibiting FABP1, and knocking down FABP1 alleviated ferroptosis of HUVEC cells, we subsequently investigated whether BSHXP improved HUVEC cell injuries by regulating FABP1-mediated ferroptosis. Our research results displayed that BSHXP significantly downregulated the Fe^2+^ (Figure 7A), ROS (Figure 7C), IL-1β, IL-6, MCP-1, and VCAM-1 (Figures 7E–H) levels, apoptosis rate (Figure 7I), and ACSL4 and 4-HNE (Figure 7D) protein expression levels of HUVEC cells, and upregulated the GSH level (Figure 7B) as well as GPX4 and SLC7A11 (Figure 7D) protein expression levels of HUVEC cells. In addition, overexpressing FABP1 exerted the same effects as erastin, both of which effectively reversed the effects of BSHXP on the indicators mentioned above. The above results implied that BSHXP improved HUVEC cell injuries by suppressing FABP1-mediated ferroptosis.

## 4 Discussion

AS is the leading cause of cardiovascular disease incidence and mortality (Burnett et al., 2020). At present, the clinical treatment of AS mainly focuses on lipid regulation and plaque stabilization, and statins are the first choice for treating AS (Zhang et al., 2023a). However, long-term use of statins causes adverse reactions, such as elevated liver enzymes, liver damage, and muscle pain (Liu et al., 2019). Ox-LDL is associated with lipid formation and is an important characteristic of vulnerable plaques (Bentzon et al., 2014). In previous studies, ox-LDL-induced HUVEC cell injuries were extensively applied to simulate AS *in vitro* (Bian et al., 2020; Wang et al., 2022b). Therefore, this study also constructed an ox-LDL-induced HUVEC cell injury model to investigate the effects of BSHXP on AS. First, we adopted the network pharmacology approaches to predict the core metabolites, hub targets, and therapeutic pathways of BSHXP. In this study, 12 core metabolites, 10 hub targets, and related therapeutic pathways were predicted, based on which the subsequent studies were performed.

Lipid metabolism disorder is the pathological basis of AS. FABP promotes the transport and diffusion of long-chain fatty acids and long-chain fatty acyl CoA and esterifies them to enhance the secretion of low-density lipoprotein (Atshaves et al., 2010). Meanwhile, FABP1 is one downstream target of the PPAR pathway predicted by the above enrichment analysis. In addition, the excessive activation of SREBP2, a cholesterol sensor in the endoplasmic reticulum, leads to its inability to metabolize lipids, eventually resulting in significant fat accumulation and a series of subsequent inflammatory reactions (Benatzy et al., 2024). Therefore, this study first investigated whether BSHXP regulated the lipid metabolism of HUVEC cells by regulating FABP1 and SREBP2. It was indicated that the expression level of FABP was upregulated in ApoE (−/−) AS mice (Ju et al., 2024). Moreover, another study indicated that ginkgolide B downregulated ox-LDL-induced altered expression of SREBP2 in HUVEC cells (Wang et al., 2019). In this study, we discovered that ox-LDL dramatically elevated the lipid droplet content and FABP1 and SREBP2 protein expression levels of HUVEC cells, which was consistent with previous studies mentioned above. After excluding the toxic dosage of BSHXP, we affirmed that BSHXP effectively reduced these indicators in a dose-dependent manner. In addition, knocking down FABP1 also significantly reduced lipid droplet content and SREBP2 protein expression level of HUVEC cells. Our study indicated that BSHXP regulated FABP1-mediated lipid metabolism. In addition, one previous study found that the downregulation of the peroxisome proliferator-activated receptor α (PPARα) signaling pathway reduced FABP1 expression to regulate ferroptosis (Wu et al., 2022). Meanwhile, another previous study PPARα targets SREBP2 to regulate the biosynthesis of cholesterol (Liu et al., 2022b). Therefore, BSHXP might regulate PPARα to inhibit FABP1 and SREBP2 to suppress ferroptosis.

Cellular iron ions promote further hydroxylation of unsaturated fatty acids and the production of large amounts of lipid peroxides and hydroperoxides through the Fenton reaction, inducing ferroptosis (Stockwell et al., 2020). In recent years, more and more evidence has shown that ferroptosis, characterized by iron accumulation, redox imbalance, and lipid peroxide accumulation, was involved in the development of AS (Lin et al., 2021; Xu et al., 2024). GPX4 is an important pathway for anti-lipid peroxidation, SLC7A11 is a component of the cystine/glutamate reverse transport system to provide substrates for GSH, and ACSL4 acylates arachidonic acid and adrenal acid to promote lipid peroxidation (Chen et al., 2021). Lipid peroxidation is the ultimate executor of ferroptosis, and the level of 4-HNE could reflect the severity of ferroptosis (Zhang et al., 2023b). Therefore, this study subsequently investigated whether BSHXP alleviated ox-LDL-induced HUVEC cell injuries by regulating ferroptosis. One previous study indicated that berberine stabilized atherosclerotic plaque by activating the SLC7A11/GPX4 pathway to suppress ferroptosis, accompanied by reduced ROS and Fe^2+^ levels (Wang et al., 2024). Another study demonstrated that overexpressing KLF7 attenuated endothelial cell injury by suppressing ACSL4-mediated ferroptosis, accompanied by elevated GSH levels and reduced 4-HNE expression (Xiong et al., 2024). In this study, we confirmed that BSHXP reduced Fe^2+^ and ROS levels as well as ACSL4 and 4-HNE expression levels, and elevated GSH levels and SLC7A11 and GPX4 expression levels, which were consistent with the previous studies mentioned above. Meanwhile, our studies affirmed that knocking down FABP1 exerted the same effects as BSHXP on ferroptosis, and overexpressing FABP1 and administering erastin effectively reversed the effects of BSHXP on ferroptosis. Our studies suggested BSHXP suppressed FABP1-mediated ferroptosis of HUVEC cells.

Endothelial injury is an important link in cardiovascular disease. Ferroptosis will destroy the iron homeostasis and antioxidant effect of cells in the vascular system, leading to endothelial destruction. After the destruction of vascular endothelial integrity, the secretion of IL-1β, IL-6, MPC-1, and VCAM-1 increases to form an inflammatory microenvironment, recruiting monocytes to the vascular injury site to form plaque to promote AS (Fan and Watanabe, 2003). In addition, ferroptosis may trigger inflammation to promote the adhesion and infiltration of monocytes by activating adhesion molecules (such as ICAM-1 and VCAM-1) and MCP-1/CCL2, further intensifying the formation of foam cells (Guo et al., 2024). Therefore, this study finally investigated whether BSHXP alleviated inflammation and adhesion by inhibiting ferroptosis. One previous study indicated that atorvastatin attenuated endothelial cell injury by suppressing ACSL4-mediated ferroptosis, accompanied by reduced IL-1β, IL-6, and VCAM-1 (Tan et al., 2024). In addition, the injection of human ferritin heavy chain effectively attenuated the AS process by inhibiting ferroptosis, accompanied by the decline of MCP-1 (Yuan et al., 2023). We affirmed that BSHXP prominently reduced IL-1β, IL-6, VCAM-1, and MCP-1 levels and the apoptosis rate of HUVEC cells. Meanwhile, overexpressing FABP1 and erastin effectively reversed the effects of BSHXP on the above indicators of HUVEC cells. Our results proved that BSHXP alleviated HUVEC cell injuries, including inflammation, adhesion, and apoptosis, by suppressing FABP1-mediated ferroptosis.

Despite the mechanistic findings of this study, several limitations should be acknowledged. First, the current data were generated from an *in vitro* model using ox-LDL-treated HUVECs, and the absence of *in vivo* validation limits translational applicability. Second, although the study revealed FABP1-mediated lipid metabolism as a key pathway, the analysis of lipid metabolic changes was relatively superficial and lacked untargeted metabolomic profiling. Third, the specific bioactive metabolites responsible for the observed effects remain unidentified, and the multi-component nature of BSHXP complicates mechanistic interpretation. To address these limitations, future studies might focus on several directions. Animal models of atherosclerosis may be employed to validate the anti-ferroptotic and anti-inflammatory effects of BSHXP under physiological conditions. Untargeted metabolomics and lipidomics will allow a comprehensive evaluation of its impact on lipid pathways. Isolation and functional characterization of individual botanical drugs or metabolites are necessary to clarify their specific contributions and mechanisms of action. In addition, network pharmacology analysis suggests the involvement of multiple targets beyond FABP1; thus, further investigation into alternative therapeutic targets is warranted. In addition, studies have confirmed that regulating gut microbiota was conducive to mitigating various cardiovascular diseases, including AS (Chen et al., 2024; Witkowski et al., 2020). It has been affirmed that multiple botanical drugs in BSHXP could regulate gut microbiota, such as *Polygonatum cyrtonema* Hua, *Lycium barbarum* L., *S. miltiorrhiza* Bunge., and *C. pinnatifida* Bge. (Jiang et al., 2024; Li et al., 2023; Cai et al., 2024; Guo et al., 2021), indicating a possible role in host-microbe interaction that merits exploration. Meanwhile, some previous studies have demonstrated that some long non-coding RNAs (lncRNAs) and microRNAs (miRNAs) also participated in regulating ferroptosis, such as lncRNA AGAP2-AS1 and miR-522 (Huang et al., 2023; Zhang et al., 2020). Non-coding RNAs may also be involved in ferroptosis regulation and could serve as epigenetic targets of BSHXP. Previous studies affirmed that the application of some nanozymes, nanocatalysts, and nanobodies contributed to further regulating ferroptosis (Zhu et al., 2025; Zhu et al., 2024; Li et al., 2021). Advances in nanotechnology, including nanozyme or nanobody-assisted delivery, may enhance the precision and efficacy of botanical-based ferroptosis interventions. Moreover, statins have been extensively used for AS treatment in the clinic, which block the intracellular hydroxymevalproic acid metabolic pathway to reduce the intracellular cholesterol synthesis through inhibiting the endogenous rate limiting-enzyme of cholesterol synthesis competitively, HMG CoA reductase (Sirtori, 2014). Therefore, BSHXP may possess synergistic effects with statins. However, there are still no relevant studies to investigate the synergistic effects of them. Further investigations of the synergistic effects and related mechanisms between BSHXP and statins will contribute to the clinical treatment of AS. In addition, it was proved that the crosstalk between cells and microenvironment was also associated with alterations in iron and lipid metabolism (Li et al., 2025). Notably, although BSHXP reduced intracellular Fe^2+^ levels, it remains unclear whether this is due to increased iron efflux, modulation of iron transporters (e.g., transferrin, DMT1), or the presence of iron-chelating phytochemicals. Given that polyphenols in botanical formulas may exhibit intrinsic metal-binding capacity, further investigation using cyclic voltammetry and electron paramagnetic resonance spectroscopy is recommended to determine whether BSHXP contains functional iron chelators contributing to its anti-ferroptotic effects.

## 5 Conclusion

In this study, we predicted 12 core metabolites, 10 hub targets, and related therapeutic pathways of BSXHP. We proved that BSHXP could reduce FABP1 expression level, regulate lipid metabolism, inhibit ferroptosis, and decline inflammation, adhesion, and apoptosis of HUVEC cells. Knocking down FABP1 could also regulate lipid metabolism and suppress lipid metabolism. In addition, overexpressing FABP1 and erastin effectively reversed the effects of BSHXP on the ferroptosis, inflammation, adhesion, and apoptosis of HUVEC cells. In conclusion, BSHXP inhibited the ferroptosis of HUVEC cells by regulating FABP1-mediated lipid metabolism.

## Data Availability

The data presented in the study are deposited in the Harvard Dataverse repository, DOI: 10.7910/DVN/X049CQ, available at: https://dataverse.harvard.edu/dataset.xhtml?persistentId=doi:10.7910/DVN/X049CQ.

## Glossary

AS: Atherosclerosis
BSHXP: Bushen Huoxue Prescription
HUVEC: Human Umbilical Vein Endothelial Cell
ox-LDL: Oxidized Low-Density Lipoprotein
FABP1: Fatty Acid Binding Protein 1
SREBP2: Sterol Regulatory Element-Binding Protein 2
ROS: Reactive Oxygen Species
GSH: Glutathione
GPX4: Glutathione Peroxidase 4
ACSL4: Acyl-CoA Synthetase Long Chain Family Member 4
4-HNE: 4-Hydroxy-2-Nonenal
SLC7A11: Solute Carrier Family 7 Member 11
IL-1β: Interleukin-1 Beta
IL-6: Interleukin-6
MCP-1: Monocyte Chemoattractant Protein-1
VCAM-1: Vascular Cell Adhesion Molecule-1
MTT: Methylthiazolyldiphenyl-Tetrazolium Bromide
ELISA: Enzyme-Linked Immunosorbent Assay
qRT-PCR: Quantitative Real-Time Polymerase Chain Reaction
WB: Western Blot
UHPLC-Q/Exactive: Ultra-High Performance Liquid Chromatography-Quadrupole/Exactive Mass Spectrometry
PPI: Protein-Protein Interaction
GO: Gene Ontology
KEGG: Kyoto Encyclopedia of Genes and Genomes
TCMSP: Traditional Chinese Medicine Systems Pharmacology Database
DAVID: Database for Annotation, Visualization and Integrated Discovery
STRING: Search Tool for the Retrieval of Interacting Genes/Proteins
NC: Negative Control
oe-FABP1: Overexpression of FABP1
sh-FABP1: Short Hairpin RNA for FABP1
NRF2: Nuclear Factor Erythroid 2-Related Factor 2
PPAR: Peroxisome Proliferator-Activated Receptor
